# Walking Instability in a Patient with Known Neuro-Behçet’s Disease

**DOI:** 10.31138/mjr.31.1.78

**Published:** 2020-03-31

**Authors:** Styliani Partalidou, Vasiliki Tsiakalou, Ioannis Vassilakos, Dimitrios Kassimos

**Affiliations:** 1 Military School, 401 General Hospital of Athens, Greece,; 2 Rheumatology Department, 401 General Hospital of Athens, Greece

**Keywords:** Neuro-Behçet’s Disease, rhombencephalitis

## Abstract

A 53-year-old man with known history of Neuro-Behçet›s Disease (NBD) presented to the Emergency Department with numbness on the left side of the body and the face. The patient was admitted to the Neurological Department and after a thorough investigation, the magnetic resonance imaging (MRI)of the brain revealed a lesion on the brainstem (rhombencephalitis). The case is presented due to rarity of the clinical picture and the good outcome. The therapeutic strategy and the modification of his medication is discussed.

## INTRODUCTION

Behçet’s disease is a variable vessel vasculitis of unknown etiology that presents with oral and genital ulcers and uveitis. The disease can also present with skin lesions, musculoskeletal problems, Central Nervous System (CNS) involvement, gastrointestinal disorders and major vessel involvement.^1^ The majority of cases come from areas around the Mediterranean and Japan. The prevalence has been reported to less than 1/10^5^ (North and Central Europe) to 20/10^5^ in Japan, China and Korea.^1^ Turkey comes in the first place with 20–420 per 100.000 population.^2^ Neurological involvement is estimated about 3%–9% of the cases and it is considered as a bad prognostic factor of increased mortality.^1^ The diagnosis is based on clinical evaluation as no test can prove the presence of the disease.

## CASE DESCRIPTION

A 53-year-old Greek policeman with known history of NBD since 2008 under Cyclosporin-A (CSA) and colchicine, with oral ulcers, skin lesions (pseudofolliculitis) and an episode of CNS involvement (numbness on the right side of the body and the face), presented to the Emergency Department with recent onset of numbness on the left side of the body and the face, the jaw in particular. A thorough clinical evaluation was made by both a neurologist and a cardiologist which did not reveal anything abnormal. His vital signs, full blood count (FBC), liver blood tests (LBT), urea, creatinine, electrolytes (U&E), C-reactive protein (CRP) and troponin were within normal limits. The electrocardiograph (ECG) showed normal sinus rhythm with no ST disorders. A computed tomography (CT) of the brain was performed and it was normal, as well. Since a possible stroke and an acute myocardial infraction (AMI) were excluded, the patient was discharged with the recommendation of conducting an MRI of the brain and the cervical spine. However, two days later, he returned to the outpatient Neurological Department following symptoms and signs listed in *Table 1*.

**Table 1.** Clinical symptoms and signs of the patient.

**CLINICAL SYMPTOMS**	**CLINICAL SIGNS**
Gait instability	Bilateral instability in gait
Blurred vision	At the examination of cerebellum there was tremor and weakness of the left hand
Numbness on the left side of the body and the face	The left knee reflex was not reproduced

Differential diagnosis included a flare of the disease, multiple sclerosis (MS), primary vasculitis of the CNS, neurosarcoidosis, CNS tuberculosis and tumors (primary lemphoma, brainstem glioma).^1^ The patient, then, was admitted in the Neurological Department with possible diagnosis of Behçet’s vasculitis and received five-days pulse of 1gr methylprednisolone intravenously (IV), followed by per os (p.o.) prednisone at the dose of 1mg/kg/day and anti-osteoporotic medication (calcium supplements, vitamin D, alendronate). During the current admission, FBC, LBT, U&E, CRP, erythrocyte sedimentation rate (ESR) and immunological exams were within normal limits, as shown in *Table 2*.

**Table 2.** Laboratory results before and throughout the admission.

	**ED**	**2^nd^ attendance**	**Deterioration day**	**Exit day**
WBC(K/μL)/neutrophils (%)	7,9/59	8,2/51,3	7,5/60	7,3/58
HT(%)/Hb(g/dL)	45,7/15	44/15,3	44,2/15	44,5/14,8
SGOT(U/L)SGPT(U/L)	14/17	16/17	15/18	20/22
Urea(mg/dL)/Creatinine(mg/dL)	58/1	55/1,1	44/0,9	48/1
CRP(mg/L)/ESR	3,2/15	3,3/14	3,1/10	2,8/9

**Figure 1.**
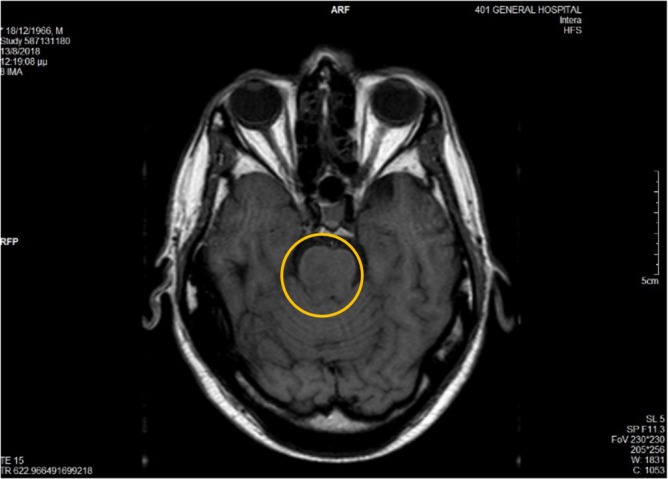
T2 section.

**Figure 2.**
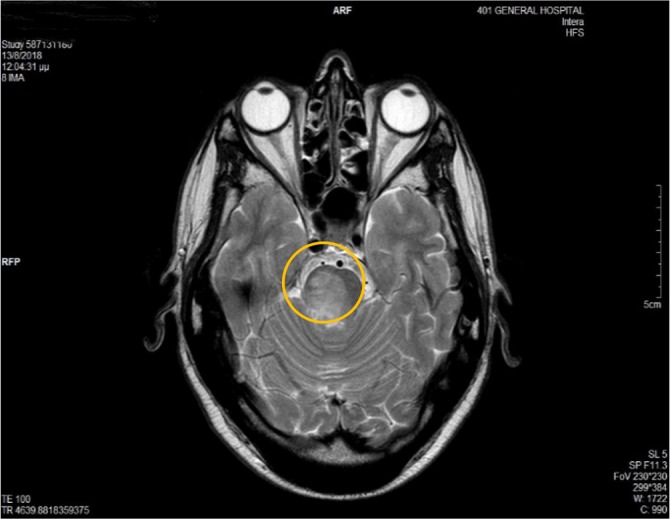
T1 section.

**Figure 3.**
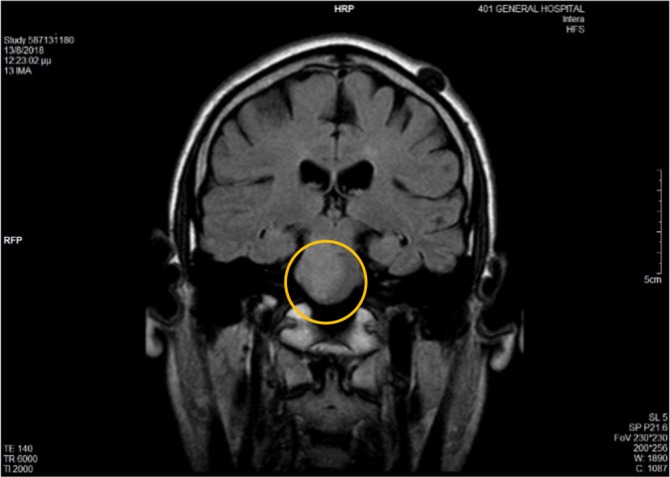
FLAIR.

Magnetic Resonance Angiograph (MRA), ultrasound (U/S) of carotids and spinal arteries, MRI of the cervical spine and retinoscopy were normal. However, MRI of the brain revealed a lesion on the brainstem that appeared with high signal on T2 and FLAIR sections and equal signal on T1 sections, as demonstrated in *Figure 1*, *Figure 2*, and *Figure 3.*

This finding was highly indicative of an NBD flare, but it could not exclude categorically other conditions.^3^ About seven days after the admission, the instability deterioreted while presenting Babinski and Barre sign on the left side. Then, a lumbar puncture was conducted which showed slightly higher white blood cells (20 cells/μL). The albumin and pressure of the cerebrospinal fluid (CSF) were normal while oligoclonic zones and culture were negative. The patient was also found positive on the test for HLA-B51. After discussion with the specialists, it was proposed to stop CSA due to its neurotoxic effects and replace it with another agent, such as azathioprine (AZA), cyclophosphamide (CYC), mycophanolate acid (MMF) or a biological agent, such as anti-TNF (infliximab or adalimumab) and continue steroids, methylprednisolone, at the dose of 16mg twice a day p.o.^4^ Based on the latest recommendations of EULAR for the treatment of Behçet’s disease, AZA was preferred at the dose of 100mg^5^. Four days after the initiation of AZA, the patient was discharged, apparently having improved. The prednisone was gradually tapered. Four weeks later, the patient visited the outpatient Rheumatology Department for re-evaluation. His recovery was very good, there was no neurological deficit whatsoever, and he continued his work with some difficulties.

## DISCUSSION

Neurological involvement is estimated in about 3%–9% of the cases, and it is considered as a bad prognostic factor of increased mortality.^1^ It is also more frequent in males (3:1).^6^ There are two patterns: parenchymal that mostly affects the brainstem and the hemispheres, and the vascular that includes dural sinus thrombosis.^7^ The clinical spectrum includes every neurological manifestation from both CNS and peripheral nervous system (PNS), although the latter is less frequent.^2^ The patient had a rare manifestation, with sensor disorder at first. The similarity in the two episodes with an interval of 10 years is remarkable, as is the fact that the patient was under CSA despite the contraindication for NBD.^5^ However, even under this medication he was in remission for a long period, that is 10 years. It should also be pointed out that the most common cause of rhombencephalitis is infection of *Listeria Monocytogenes*, followed by Behçet’s disease.^8^ Other causes of rhombencephalitis are viral infections such as enteroviruses (enterovirus 71, common), flaviviruses (Japanese encephalitis, common) and herpes viruses (uncommon), autoimmune diseases other than Behçet’s Disease, such as SLE and relapsing polychondritis (both rare)and lymphoma (rare).^8^ Since the culture of CSF was negative, and given the history of the patient, a flare of the NBD was the most suitable diagnosis in this case. As for the treatment, it is known that CSA is contraindicated in NBD due to its neurotoxic effects, hence, this was the basic step for the patient’s improvement.^4^ Another remarkable point is the fact that the patient is totally functional with no neurological deficits and he is able to continue his demanding job.
